# Aids to Postgraduate Medicine

**Published:** 1984-01

**Authors:** 


### Book Review

**AIDS TO POSTGRADUATE MEDICINE**
**J. L. Burton**
**Churchill Livingstone, 1983**
**pp. 232; £4.50, 4th Edition**

The fact that this handy paper back has now reached its fourth edition in 13 years is a tribute to its popularity and the energy of the author. It gives in note form causes of various medical conditions, their diagnosis and management. It is a pity that only gonorrhoea and syphilis are included in venereology and no mention is made of N.S.U., L.G.V., scabies, A.I.D.S., etc. Another deficiency is tropical and infectious disease: I think it is wrong to devote a whole page to glycogen storage disease and none to the eminently treatable meningitis and malaria.

The weakness of any book which relies on lists, e.g. 14 causes of ectopic humoral syndromes and 16 causes of paraplegia, is that the reader is given little guidance on frequency or importance with regard to management and treatment. Perhaps the title of the book should be Aids to Passing the M.R.C.P., in which case there should be some pages on geriatrics, paediatrics, psychiatry and clinical pharmacology. Certainly, it should be clearly stated that most candidates fail the exam because of the clinical rather than the written part. A sense of balance is also required rather than being able to recite the 11 groups of causes of cardiomyopathy.

Despite the above comments, the book is admirably clear and guides the postgraduate student in to logical thought, as for example, in listing the different mechanisms whereby coma develops. It brings together a lot of information in a white-coat-pocket size, so that quick reference in out-patients or ward rounds will be a great help in revising for the M.R.C.P.

